# Control of response interference: caudate nucleus contributes to selective inhibition

**DOI:** 10.1038/s41598-020-77744-1

**Published:** 2020-12-01

**Authors:** Claudia C. Schmidt, David C. Timpert, Isabel Arend, Simone Vossel, Gereon R. Fink, Avishai Henik, Peter H. Weiss

**Affiliations:** 1 Cognitive Neuroscience, Institute of Neuroscience and Medicine (INM-3), Research Centre Juelich, Juelich, Germany; 2 Department of Neurology, Faculty of Medicine and University Hospital Cologne, University of Cologne, Cologne, Germany; 3 Department of Psychology and Zlotowski Center for Neuroscience, Ben-Gurion University of the Negev, Beer-Sheva, Israel; 4 Department of Psychology, Faculty of Human Sciences, University of Cologne, Cologne, Germany

**Keywords:** Cognitive control, Human behaviour

## Abstract

While the role of cortical regions in cognitive control processes is well accepted, the contribution of subcortical structures (e.g., the striatum), especially to the control of response interference, remains controversial. Therefore, the present study aimed to investigate the cortical and particularly subcortical neural mechanisms of response interference control (including selective inhibition). Thirteen healthy young participants underwent event-related functional magnetic resonance imaging while performing a unimanual version of the Simon task. In this task, successful performance required the resolution of stimulus–response conflicts in incongruent trials by selectively inhibiting interfering response tendencies. The behavioral results show an asymmetrical Simon effect that was more pronounced in the contralateral hemifield. Contrasting incongruent trials with congruent trials (i.e., the overall Simon effect) significantly activated clusters in the right anterior cingulate cortex, the right posterior insula, and the caudate nucleus bilaterally. Furthermore, a region of interest analysis based on previous patient studies revealed that activation in the bilateral caudate nucleus significantly co-varied with a parameter of selective inhibition derived from distributional analyses of response times. Our results corroborate the notion that the cognitive control of response interference is supported by a fronto-striatal circuitry, with a functional contribution of the caudate nucleus to the selective inhibition of interfering response tendencies.

## Introduction

Control of response interference refers to a subprocess of cognitive control that demands to resolve a response conflict by inhibiting a prepotent yet inappropriate response tendency in favor of selecting a task-appropriate response^1^. Conceptually, response interference control is a selective inhibition process that serves to suppress the activation of one response (but not another) and thus prevents an incorrect response selection due to interfering response tendencies^2^. Other conceptually different cognitive components of conflict-driven (inhibitory) control are cognitive flexibility, working memory, and (global) response inhibition^3,4^. Note that the term (global) response inhibition is used in the current study to describe the control processes of withholding a prepotent or canceling an already initiated yet inappropriate action, which may imply globally suppressing any ongoing responses^5^.

There is general agreement that the diverse cognitive control (sub)processes (i.e., response interference control, response inhibition, cognitive flexibility, and working memory) rely on functionally connected (pre)frontal cortical regions^6,7^. More specifically, the anterior cingulate cortex (ACC) has been implicated in the detection of (response) conflicts and the monitoring of control demands^8,9^. In contrast, the dorsolateral prefrontal cortex (DLPFC) is thought to subserve the active maintenance of (internal) goals and/or task representations in order to implement cognitive control^10,11^. Other cortical areas that are commonly recruited by the diverse cognitive control processes include the (right) inferior frontal gyrus (IFG), the pre-supplementary motor area (pre-SMA), the insula as well as (posterior) parietal cortices^12,13^.

The functional interaction between the frontal (and parietal) brain regions during the execution of cognitive control is considered to be modulated by subcortical structures, such as the basal ganglia (BG) and thalamus^14,15^. While recent neuroimaging meta-analyses reported increased activity in the dorsal striatum (the main input structure of the BG comprising the caudate nucleus and putamen) and thalamus across a wide range of cognitive control demands^16–18^, only (global) motor response inhibition consistently activated the bilateral thalamus^16^. Besides the latter finding, previous work has emphasized the role of the subthalamic nucleus (STN) in (global) response inhibition^19–21^. In contrast, the dorsal striatum (particularly the caudate nucleus) has mainly been associated with working memory^22,23^, cognitive flexibility/task-switching^24,25^, and (associative) control-learning mechanisms in response to changing control demands^26,27^. Besides, there is growing evidence for relevant contributions of the dorsal striatum to the control of response interference (including selective inhibition). In particular, the electrophysiological recording of neural activity in the caudate nucleus of non-human primates performing an anti-saccade task has revealed evidence for the dorsal striatum’s functional relevance for selective inhibitory control^28,29^. Moreover, these studies proposed that striatal neurons projecting to the BG’s direct pathway promote volitional saccades away from a visual stimulus (i.e., anti-saccades). In contrast, striatal neurons projecting to the BG’s indirect pathway suppress reflexive saccades toward the stimulus in favor of an anti-saccade^30,31^. Likewise, drawing upon models of BG functions, recent neuro-computational studies provided further support for the notion that the dorsal striatum might mediate selective inhibition via the indirect pathway of the BG, as studied with anti-saccade, saccade-override, and Simon tasks^32,33^. In patients with neurodegenerative disorders affecting the striatum, such as Parkinson’s disease (PD) and Huntington’s disease (HD), experimental-psychological studies found impairments in resolving response interference in Simon and arrow-versions of the Eriksen flanker tasks^34–36^, including deficits in the selective inhibition of interfering response tendencies^37,38^. Finally, some previous functional neuroimaging findings in healthy subjects also point to the dorsal striatum’s involvement in response interference control in (variants of) the Simon task^39,40^. Notably, these latter studies do not allow to infer whether the dorsal striatum specifically contributed to selective inhibition or whether it was involved in other processes required for interference control.

Accordingly, the current study examined the neural mechanisms subserving the control of response interference further. Using event-related functional magnetic resonance imaging (fMRI) in healthy subjects, we specifically focused on the role of the dorsal striatum in the selective inhibition of interfering response tendencies.

To investigate the control of response interference (including selective inhibition), we here applied the Simon task in which participants are asked to respond to a predefined stimulus feature (e.g., color or shape) based on an arbitrary mapping to left or right manual responses, irrespective of the spatial location of the stimulus^41^. In each trial, the stimulus randomly appears either on the left or on the right side of a fixation point and hence the stimulus location either spatially match or mismatch the task-assigned (correct) response. A mismatch between the spatial location of the stimulus and the relative position of the assigned response (i.e., an incongruent condition) is typically associated with longer response times (RTs) and increased error rates, as compared to when both positions correspond (i.e., a congruent condition). Accordingly, even though the stimulus location is irrelevant for the task, it facilitates the response to the task-relevant stimulus feature in congruent trials but interferes with the correct response selection in incongruent trials^42^. The RT difference between incongruent and congruent conditions is termed the Simon effect^43^ and used as a measure of the ability to resolve stimulus–response conflicts due to interfering response tendencies in incongruent trials^44^.

In the present study, a unimanual version of the Simon task was applied in which left or right responses were given with the index and middle fingers of the same hand (see below). A unimanual response set-up was used here to relate the current functional imaging results to our previous study in stroke patients that investigated response interference control processes after unilateral lesions of the striatum^45^. Specifically, by using a unimanual task in that previous study we aimed to avoid confounding effects of potential paresis of the contralesional hand or arm following stroke. Motion direction of (coherently) upward or downward moving dots was defined as the task-relevant stimulus feature since motion stimuli might be more sensitive to engage subcortical structures^46,47^.

Resolving stimulus–response interference has been discussed in terms of demanding the selective inhibition of interfering response tendencies during response selection^48,49^. While there are also other approaches used to explain how stimulus–response interference is controlled (e.g., via attentional control processes)^50^, we here relied on the *activation-suppression hypothesis* that accounts for the temporal dynamics of a proposed inhibitory control process in conflict tasks and particularly in the Simon task^51^. According to this hypothesis, an (incorrect) response activation (i.e., a response tendency evoked by the task-irrelevant stimulus location) is followed by selective inhibition in incongruent trials to execute the correct response. Since the process of (selective) inhibition gradually builds up within a trial, its impact is more apparent in slower than in faster responses. Given these dynamics, the Simon effect is affected by selective inhibition more in trials with relatively long (intra-individual) RTs than in trials with shorter RTs and consequently decreases with slower responses. The process and individual efficiency of selective inhibition can be revealed by RT distributions in which the Simon effect is analyzed as a function of response latency^49^. Specifically, a (more) decreasing Simon effect across the RT distribution (i.e., with slower responses) is associated with (more efficient) selective inhibition of interfering response tendencies^52^. Previously, the RT distribution parameter of selective inhibition (i.e., the decrease of the Simon effect across the RT distribution) has already been proven applicable for relating inter-individual differences in selective response inhibition to the underlying neural mechanisms^53,54^ as well as for examining group differences in the efficiency of selective inhibition between neurological patients with assumed impairments in inhibitory control and healthy subjects^37,45^.

As outlined above, models and studies of basal ganglia function propose that—at the subcortical level—response selection and selective inhibition may be modulated by the dorsal striatum through a balance of activity in the direct and indirect BG pathways, respectively^33,55^. Accordingly, the dorsal striatum (i.e., caudate nucleus and putamen) should be involved in the control of response interference by putatively mediating the selection of task-appropriate responses and/or the selective inhibition of interfering response alternatives. In line with this assumption, our previous study in stroke patients indicated less efficient selective inhibition to resolve response interference after unilateral lesions of the striatum when taking into account the temporal dynamics of the interference control processes across the RT distribution^45^. Hence, we here hypothesized that the individual efficiency of selective inhibition as indexed by the decrease of the Simon effect across the RT distribution^49^ is associated with (increased) activation of the dorsal striatum.

## Material and methods

### Participants

Initially, 16 healthy subjects participated in the study. All participants were screened for factors contraindicating magnetic resonance imaging (MRI) scanning, provided written informed consent to participate in the study, and received financial compensation for their participation.

Three participants had to be excluded from further analyses due to extensive head movement (rotation > 3°, *n* = 1) or technical problems (*n* = 2) during scanning. Thus, the final sample included 13 subjects (9 female) with a mean age of 25.5 years (*SD* = 4.2 years). All subjects were right-handed, according to the Edinburgh Handedness Inventory (laterality quotient [LQ]: *M* = 92.4, *SD* = 11.0)^56^, and reported normal or corrected-to-normal visual acuity. None of the participants suffered from any neurological or psychiatric diseases.

The local ethics committee of the Faculty of Medicine of the University of Cologne had approved the study, which was conducted following the ethical principles of the World Medical Association (Declaration of Helsinki; revised version, October 2013).

### Stimuli and task

The software Presentation® (Neurobehavioral Systems, Inc.) was used for stimulus presentation and response logging.

The task was presented on a screen (screen width: 65 cm) mounted on the wall at the back of the magnet bore. Participants viewed the monitor via a movable mirror system attached to the MR head coil (viewing distance: 245 cm).

The current unimanual Simon task resembles the task design used in our previously published study on response interference control and striatal lesions^45^. The target stimulus consisted of a square involving a flow field of dots coherently moving either upward or downward. The squares (subtending 2° × 2° of visual angle) were displayed on a black background either to the left or to the right side of a white centrally presented fixation cross and positioned such that their boundaries were 2° left or right of the fixation point.

**Figure 1:**
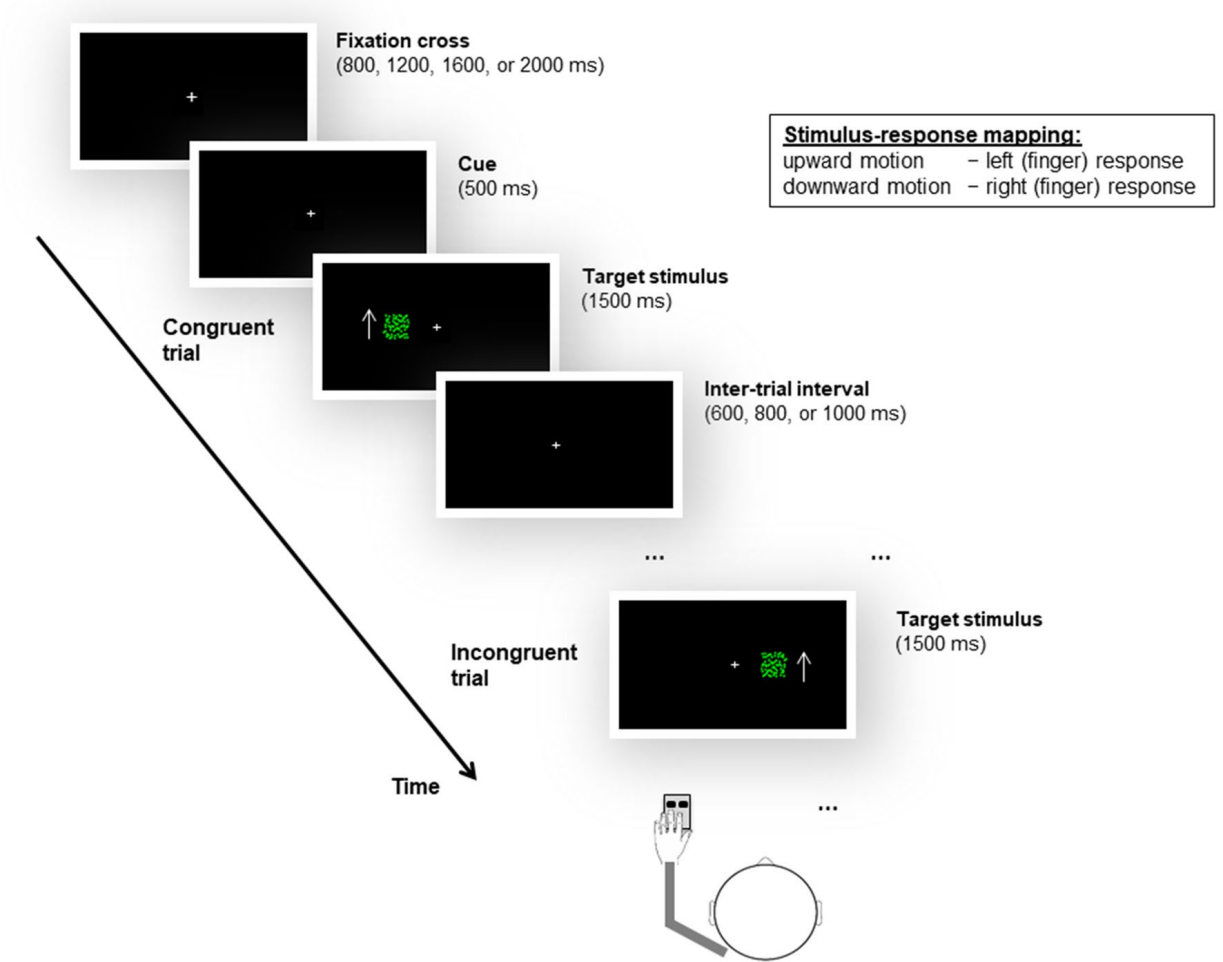
Schematic of the unimanual Simon task. Participants were required to discriminate the motion direction of a moving dots stimulus by responding with the index or middle finger of the same hand, irrespective of the spatial location (i.e., left or right side of the fixation cross), at which the stimulus was presented. Each participant completed two experimental blocks by successively using the left and right hand. The example here shows a left-hand response condition in which upward-moving dots are mapped to a left response (i.e., the middle finger) and downward-moving dots are mapped to a right response (i.e., the index finger). Based on the spatial correspondence between the stimulus position (left or right side of the fixation cross) and the task-assigned (relative) response side (left or right finger), a stimulus with upward-moving dots that is presented in the left visual field represents a congruent trial. Conversely, a stimulus with upward-moving dots that appears in the right visual field is defined as an incongruent trial. Please note that the arrow was not presented to the participants during the experiment but is shown here to illustrate the motion direction of the dots. Further note that the stimulus itself did not move in position (i.e., up or down) but stayed fixated.

For the current version of the Simon task, participants were instructed to respond to the moving dots’ motion direction by giving left or right finger responses (see below), irrespective of the location at which the stimulus was presented. Based on the spatial correspondence between the position at which the stimulus appeared (i.e., left or right visual field) and the relative side of response according to the task instruction (i.e., left or right finger), two trial types were defined. A stimulus whose spatial location matched the side of response assigned by the task represented a *congruent* trial (e.g., a stimulus with upward-moving dots requiring a left finger response was presented on the left side of the fixation cross). Conversely, a stimulus whose spatial location and task-assigned response side did not match defined an *incongruent* trial (e.g., a stimulus with upward-moving dots requiring a left finger response appeared on the right side of the fixation cross; Fig. 1).

### Design and procedure

Before the functional imaging, all participants practiced the task on a laptop computer outside the MR scanner to get familiarized with the task instructions and the stimulus–response mapping.

For the mapping between the task-relevant stimulus feature (i.e., upward or downward moving dots) and the relative side of response, participants were required to use the index and middle fingers of one hand, i.e., of either the left *or* the right hand. In other words, when responding with the left hand, left responses were given with the middle finger, and right responses were given with the index finger. Conversely, participants gave left responses with the index finger and right responses with the middle finger when responding with the right hand. During the functional imaging session, each participant successively responded with the left and right hand. For response registration, MR compatible LUMItouch response keypads were used. The response keypads were always positioned to the left side (for the left-hand response condition) or the right side (for the right-hand response condition) of the participants.

The mapping between the task-relevant stimulus feature (i.e., upward or downward motion) and side of response (left or right finger) was counterbalanced across subjects. However, it was held constant within participants throughout the functional imaging experiment, i.e., independent of whether they responded with their left or right hand in the respective functional run. Half of the participants were instructed to respond to upward-moving dots by using the left finger (i.e., the middle finger of the left hand or the index finger of the right hand) and to downward-moving dots by using the right finger (i.e., the index finger of the left hand or the middle finger of the right hand). Conversely, the other half of the participants were instructed to respond to upward-moving dots with the right finger and downward-moving dots with the left finger.

The duration of the trials was jittered between 3400 and 5000 ms. More precisely, at the beginning of each trial, a central fixation cross, which remained present throughout the task, changed its size after a variable period of 800, 1200, 1600, or 2000 ms. The change in the size of the fixation cross served as a cue (for 500 ms) to signal the target stimulus’s start. Subsequently, a patch of moving dots (with either upward or downward motion) was randomly displayed at the left or right side of the fixation cross for a fixed duration of 1500 ms. Following a variable inter-trial interval (ITI) of 600, 800, or 1000 ms, the next trial started (Fig. 1). Throughout the experiment, participants were asked to maintain central eye fixation and respond as quickly and accurately as possible to the target stimuli.

Within the scanning session, each participant completed two experimental runs, separated by a short break in which participants were instructed to change their responding hand. Each run contained 160 experimental trials (i.e., 80 congruent trials and 80 incongruent trials) presented in the left or right visual field in a pseudo-randomized order. To increase the statistical efficiency of the event-related design^57^ and avoid (too) long inter-trial intervals that might reduce the magnitude of the (overall) Simon effect^58^, null trials were not included.

The duration of the fMRI experiment (both runs) amounted to approximately 35 min in total.

### Functional imaging data acquisition and preprocessing

Imaging data were acquired on a 3-T MRI system (Magnetom Trio; Siemens, Erlangen, Germany).

Functional T2*-weighted blood oxygenation level dependent (BOLD) signal sensitive images were obtained from a gradient-echo planar imaging (EPI) sequence (echo time [TE] = 30 ms; repetition time [TR] = 2200 ms; flip angle = 90°; field of view [FOV] = 200 mm × 200 mm; matrix size = 64 × 64; voxel size = 3.1 mm × 3.1 mm × 3.1 mm; bandwidth = 2232 Hz/pixel). Two functional runs were consecutively conducted within one scanning session. A total of 330 EPI volumes, each consisting of 36 axial slices covering the whole brain (slice thickness: 3.1 mm; interleaved slice acquisition, 0.3 mm gap), were collected for each subject in each of the two runs.

Additional high-resolution anatomical images (176 slices, voxel size = 1 mm × 1 mm × 1 mm) were acquired for registration purposes using a standard T1-weighted 3D magnetization-prepared rapid acquisition gradient echo (MPRAGE) sequence.

Imaging data were preprocessed and analyzed using the Statistical Parametric Mapping software package (SPM12, revision 6906, Wellcome Department of Imaging Neuroscience, London; https://www.fil.ion.ucl.ac.uk/spm/).

The first nine volumes of each run were discarded before the analysis to allow for T1 equilibration effects. The remaining volumes (2 × 321) were spatially realigned to the new first image and subsequently re-realigned to the mean of all images using a six parameter (three translations, three rotations) rigid-body transformation to correct for residual inter-scan head movements. Motion parameters were estimated for each run separately. The obtained mean EPI images for each subject (per run) were then spatially normalized to the Montreal Neurological Institute (MNI) template using the segmentation function (as implemented in SPM12). This algorithm enables combined image registration, tissue classification, and bias correction based on a probabilistic framework. Subsequently, the resulting deformation fields of the mean EPIs were applied to the individual EPI volumes and the T1 scan, which was coregistered to the mean of the realigned EPIs of the first run. Thereby, all volumes were transformed into standard stereotaxic (i.e., MNI) space, and the functional images were resampled to a 2 mm^3^ × 2 mm^3^ × 2 mm^3^ voxel size. Finally, the normalized functional images were spatially smoothed using a Gaussian kernel of 8 mm full-width at half-maximum (FWHM) to accommodate inter-participant anatomical variability.

### Statistical analysis of behavioral data

Statistical analyses of the behavioral data acquired during the fMRI scanning were performed using the software IBM SPSS Statistics (Statistical Package for the Social Sciences, Version 25, SPSS Inc., Chicago, Illinois, USA).

Trials with incorrect or missing responses were discarded before the analysis. First, error rates and mean response times (RTs) from all correct trials in each condition were analyzed separately using repeated measures analyses of variance (ANOVAs) with the within-subject factors *responding hand* (left-hand response, right-hand response), *stimulus location* (left visual field, right visual field), and *stimulus–response congruency* (congruent, incongruent). Please note that in the context of the current unimanual Simon task, the factor ‘congruency’ is defined by the intended *finger* response of one hand (i.e., left or right finger of a given hand) in relation to the stimulus location (i.e., left or right visual field).

Moreover, to assess the sequence-dependent modulation of the Simon effect^59^, mean RTs were analyzed as a function of previous and current trial congruency (after dropping the first trial in each condition) using a repeated measures ANOVA with *responding hand* (left-hand response, right-hand response), *previous stimulus–response congruency* (congruent, incongruent), and *current stimulus–response congruency* (congruent, incongruent) as within-subject factors.

Finally, to examine the process (and efficiency) of selective inhibition, RTs were further examined by distributional analyses (for each run, i.e., left-hand response condition and right-hand response condition, separately)^51,60^. First, for each participant, correct RTs were separated into congruent and incongruent conditions and sorted in ascending order. The individual RT distributions were then partitioned into four quantile bins with a roughly equal number of trials (about 20 trials per quartile), ranging from the fastest to the slowest RTs. Finally, the mean RTs were computed separately for each of the quartiles for congruent and incongruent conditions, and were analyzed using a repeated measures ANOVA with the within-subject factors *responding hand* (left-hand response, right-hand response), *stimulus–response congruency* (congruent, incongruent), and *quartile* (Q1, Q2, Q3, Q4). To further characterize a significant congruency x quartile interaction effect, a polynomial contrast was used that tests for the change/pattern (over time) of more than two group means of a dependent variable (here: for the time course of the RT difference between incongruent and congruent condition (i.e., the Simon effect) across the RT distribution)^61^. For visual depiction, the Simon effect was obtained for each quartile and, averaged across subjects, plotted against the mean RT per quartile (Vincentizing procedure)^62^.

A significance level of *p* < 0.05 was applied for all behavioral analyses.

### Statistical analysis of functional imaging data

The functional imaging data were analyzed in an event-related design using a general linear model (GLM) implemented in SPM12^63^.

At the single-subject level, the two runs (i.e., left-hand response condition and right-hand response condition) were included as separate sessions in the model (i.e., concatenated). For each run, four conditions of interest were defined, i.e., trials of upward and downward moving dots appearing in the left and right visual field, respectively (resulting in a total of eight conditions for both runs). Error trials (incorrect responses and misses) were modeled separately and included in the design matrix as a condition of no interest. The trials were modeled as events at the onset of the target stimuli, that is as delta functions with zero duration. Please note that in SPM, an ‘event’ is defined as having a stimulus duration of 0 s^64^. The resulting stimulus functions were then convolved with a canonical hemodynamic response function (HRF) and its first-order temporal derivatives to model the BOLD responses associated with the task. Additionally, the six head movement parameters derived from the (rigid-body) realignment were entered in the model as nuisance regressors to account for signals correlated with head motion. Data were high-pass filtered at 1/128 Hz to remove low-frequency signal drifts. Subsequently, parameter estimates were calculated for each voxel using a restricted maximum likelihood (ReML) approach with adjustment for serial autocorrelation by a first-degree autoregressive [AR(1)] model. Finally, eight condition-specific contrast images (four conditions of interest per run) were created for each subject.

#### Congruency effects

The individual contrast images of the first-level analysis were then used for the second-level group statistics (random-effects analysis) in a flexible factorial design with the within-subject factors *responding hand* (left-hand response, right-hand response), *stimulus location* (left visual field, right visual field), and *stimulus–response congruency* (congruent, incongruent). Correlations amongst errors and inhomogeneity of variances (non-sphericity) were estimated with restricted maximum likelihood (ReML) and adjusted for by modeling non-independence of conditions across subjects and assuming unequal variances both between conditions and between subjects.

The factorial analysis focused on the main effect of congruency using a planned *t*-contrast. In particular, to identify brain regions related to stimulus–response conflicts in the Simon task, both left and right incongruent trials were contrasted against congruent trials, collapsed across responding hand (i.e., averaged across the two runs). Imaging results from voxel-wise analyses of the factorial design are reported for activations that were significant at a statistical threshold of *p* < 0.05, family-wise error (FWE) whole-brain corrected for multiple comparisons at the cluster level using an uncorrected voxel-level threshold of *p* < 0.001^65–67^. Brain regions were identified by using the SPM Anatomy toolbox^68^ and the Automated Anatomical Labelling (AAL) atlas^69^.

Additionally, the putatively differential effect of stimulus location on congruency was investigated with planned interaction contrasts. This analysis did not reveal any significant activation clusters. To further examine the pattern of neural activity in response to incongruent versus congruent stimuli as a function of stimulus location, beta estimates from the peak voxels of the activated clusters in the main contrast of congruency were extracted for each subject. Subsequently, the mean beta estimates were subjected to separate repeated measures ANOVAs with the within-subject factors *responding hand* (left-hand response, right-hand response), *stimulus location* (left visual field, right visual field), and *stimulus–response congruency* (congruent, incongruent). As with the behavioral results (see below), there were no significant main effects of responding hand and no differential interaction effects for the left and right responding hands (all *p*-values > 0.135). Therefore, the mean beta estimates were collapsed across responding hands, and the factor stimulus location was (re)coded as ipsilateral hemifield (visual field on the side of the responding hand) and contralateral hemifield (visual field opposite to the responding hand). The mean beta estimates for congruent and incongruent trials as a function of hemifield (collapsed across responding hands) were then tested for interaction effects using a 2 × 2 repeated measures ANOVA. Again, the interaction effects of stimulus location (ipsilateral hemifield, contralateral hemifield) and congruency (congruent, incongruent) were not significant (all *p*-values > 0.181), and the extracted beta estimates are reported only descriptively (see below and Supplementary Fig. S1).

#### Individual differences in selective inhibition

Finally, to identify brain areas associated with individual differences in the efficiency of selective inhibition engaged in controlling response interference, a second-level regression analysis was performed. For this purpose, differential first-level contrast images for the main effect of congruency (incongruent > congruent) were created for each subject and entered into an fMRI regression analysis with the individual RT distribution parameter of selective inhibition (i.e., the course of the Simon effect across the RT distribution) as a covariate. That is, for each participant, the slope accounting for the course of the Simon effect across the entire RT distribution (i.e., the Simon effect values at each RT bin) was computed based on ordinary least squares (OLS) regression and included as a covariate in the second-level regression analysis. Significance testing was then performed on the covariate using a directed *t*-contrast to identify brain regions whose activation patterns co-varied with the individual’s Simon effect slope values. Based on the activation-suppression hypothesis^49^, the process (and efficiency) of selective inhibition is reflected in a decrease of the Simon effect with slower responses (i.e., a negative-going slope). Accordingly, significant activations in the fMRI regression analysis should indicate brain regions that co-varied with the individual efficiency of selective inhibition. To further probe the hypothesis that neural activation in the dorsal striatum is associated with selective inhibition (i.e., to specifically test for co-variations between activation in the dorsal striatum and the individual Simon effect slope values), a small volume correction was applied using a binary mask of the dorsal striatum (i.e., caudate nucleus and putamen, left and right combined). The mask was anatomically defined based on the AAL atlas^69^ using the WFU PickAtlas toolbox (version 3.0)^70^ available with SPM. Results from this region of interest (ROI) analysis are reported at a significance level of *p* < 0.05, FWE-corrected for the search volume. Finally, for each participant, beta estimates (for the contrast between incongruent and congruent conditions) were extracted from the peak voxels of the activated clusters in the striatum and correlated with the individual Simon effect slopes across the entire RT distribution using Pearson correlation.

## Results

### Behavioral data

Overall, participants made few errors (error rate: *M* = 1.9%, *SD* = 1.3%). The ANOVA of error rates showed a statistical trend towards a main effect of congruency [*F*(1,12) = 3.69, *p* = 0.079, η_p_^2^ = 0.24], indicating a tendency for making more errors in incongruent trials (2.4%) than congruent trials (1.5%).

Since the analyses of mean RTs did not reveal significant main effects of responding hand nor differential interaction effects for the left and right responding hand (all *p*-values > 0.229), mean RT data were collapsed across responding hands for all further analyses. Note that to this end the factor stimulus location was (re-)coded as ipsilateral hemifield (i.e., left visual field when responding with the left hand and right visual field when responding with the right hand) and contralateral hemifield (i.e., right visual field when responding with the left hand and left visual field when responding with the right hand).

**Figure 2:**
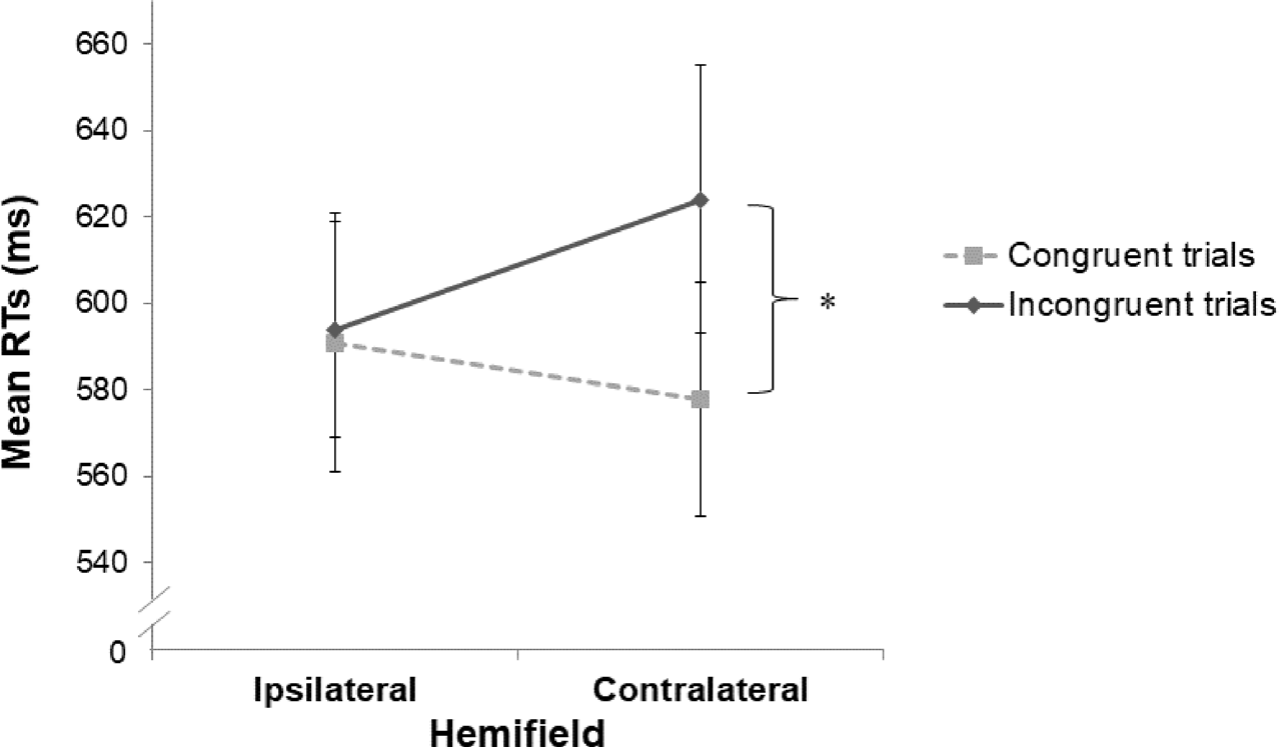
Mean response times (RTs) as a function of stimulus location and stimulus–response congruency (collapsed across responding hands) for the healthy young subjects (n = 13). Results illustrate an asymmetrical Simon effect that was more pronounced in the contralateral hemifield (for both left- and right-hand responses). Error bars indicate standard errors of the mean (SEM). *Paired-samples *t*-test: *t*(12) = 4.43, *p* = 0.001, *d* = 0.41.

There was a significant main effect of congruency [*F*(1,12) = 14.0, *p* = 0.003, η_p_^2^ = 0.54], with longer RTs for incongruent trials (609 ms) than congruent trials (584 ms). Moreover, the interaction effect of stimulus location and congruency reached significance [*F*(1,12) = 11.21, *p* = 0.006, η_p_^2^ = 0.48]. Paired-samples *t*-tests showed that the RT difference between incongruent and congruent conditions was significant for the contralateral hemifield [624 ms vs. 578 ms; *t*(12) = 4.43, *p* = 0.001, *d* = 0.41], but not for the ipsilateral hemifield [594 ms vs 591 ms; *t*(12) = 0.48, *p* = 0.641, *d* = 0.03]. Figure 2 shows the mean RTs as a function of stimulus location and stimulus–response congruency (collapsed across responding hands).

A second repeated measures ANOVA showed that the main effect of congruency was further modulated by a significant interaction effect of previous and current trial congruency [*F*(1,12) = 16.18, *p* = 0.002, η_p_^2^ = 0.57]. Post-hoc *t*-tests revealed that the RT difference between (current) incongruent and congruent conditions was absent when the preceding trial was incongruent [593 ms vs. 594 ms; *t*(12) = 0.03, *p* = 0.977, *d* = 0.002] compared to when the preceding trial was congruent [622 ms vs. 572 ms; *t*(12) = 5.32, *p* < 0.001, *d* = 0.48], reflecting a significant post-conflict behavioral adjustment as an act of (proactive) interference control^59^.

The distributional analysis of RTs furthermore yielded a significant interaction effect of congruency and quartile [*F*(3,36) = 3.61, *p* = 0.022, η_p_^2^ = 0.23], which, however, did only survive at a trend level after Greenhouse–Geisser correction accounting for non-sphericity [*F*(1.169,14.024) = 3.61, *p* = 0.074]. The subsequent polynomial contrast showed that the difference in RTs between incongruent and congruent conditions linearly decreased across the RT distribution [*F*(1,12) = 5.45, *p* = 0.038, η_p_^2^ = 0.31]. A further RT distributional analysis including ipsi- and contralateral stimuli as a separate factor revealed a significant three-way stimulus location x congruency x quartile interaction effect [*F*(1.720,20.639) = 6.74, *p* = 0.007, η_p_^2^ = 0.36; Greenhouse–Geisser corrected]. Post-hoc polynomial contrasts for ipsi-and contralateral conditions separately showed that for stimuli in the ipsilateral hemifield the difference in RTs between incongruent and congruent conditions (i.e., the Simon effect) linearly decreased with slower (intra-individual) responses and even reversed for the slowest part of the RT distribution [*F*(1,12) = 11.64, *p* = 0.005, η_p_^2^ = 0.49]. In contrast, for stimuli in the contralateral hemifield the Simon effect did not significantly differ across the RT distribution [*F*(1,12) = 0.14, *p* = 0.712, η_p_^2^ = 0.01].

### Functional imaging data

Results of the second-level factorial analysis are summarized in Table 1.

**Table 1:** Brain activation results of the factorial analysis for the effects of responding hand, visual field, and congruency (*incongruent* > *congruent*, i.e., the Simon effect).

	Hemisphere	Cluster size (voxels)	Max *T*-value	MNI coordinates		
				x	y	z
**RH > LH**						
Motor cortex	L	2581	7.88	− 48	− 20	60
Cerebellum	R	1030	8.26	16	− 48	− 20
**LH > RH**						
Motor cortex	R	650	7.60	42	− 14	68
Cerebellum	L	415	7.89	− 20	− 48	− 28
(Secondary) Somato-sensory cortex	R	439	6.43	46	− 14	14
**RVF > LVF**						
Visual cortex	L	7187	21.04	− 10	− 92	0
**LVF > RVF**						
Visual cortex	R	5381	20.43	14	− 92	6
**Incongruent > Congruent**						
Anterior cingulate cortex (ACC)	R	175	4.72	16	26	32
Caudate nucleus	R	462	4.60	20	24	6
	L		4.44	− 12	18	14
Posterior insula	R	209	4.06	50	− 16	12

For each activation cluster, a maximum with the corresponding coordinates in Montreal Neurological Institute (MNI) space, *T*-value, and the cluster extent (in the number of voxels) are given.

All activation clusters are significant at *p* < 0.05, family-wise error (FWE) corrected at the cluster level using an uncorrected voxel-level threshold of *p* < 0.001.

L = left; R = right; RH = right hand; LH = left hand; RVF = right visual field; LVF = left visual field.

Regarding the effects of responding hand, the contrasts right-hand response (RH) versus left-hand response (LH) and vice versa (LH > RH) showed significant neural activation in the respective contralateral motor cortex and ipsilateral cerebellum. Additionally, left-hand responses induced significant activation in the right (secondary) somatosensory cortex. Note that the left hand was the non-dominant hand of the participants. The main contrasts of stimulus location (i.e., visual field), namely right visual field (RVF) versus left visual field (LVF) and vice versa (LVF > RVF), significantly activated clusters in the respective contralateral visual cortices.

**Figure 3:**
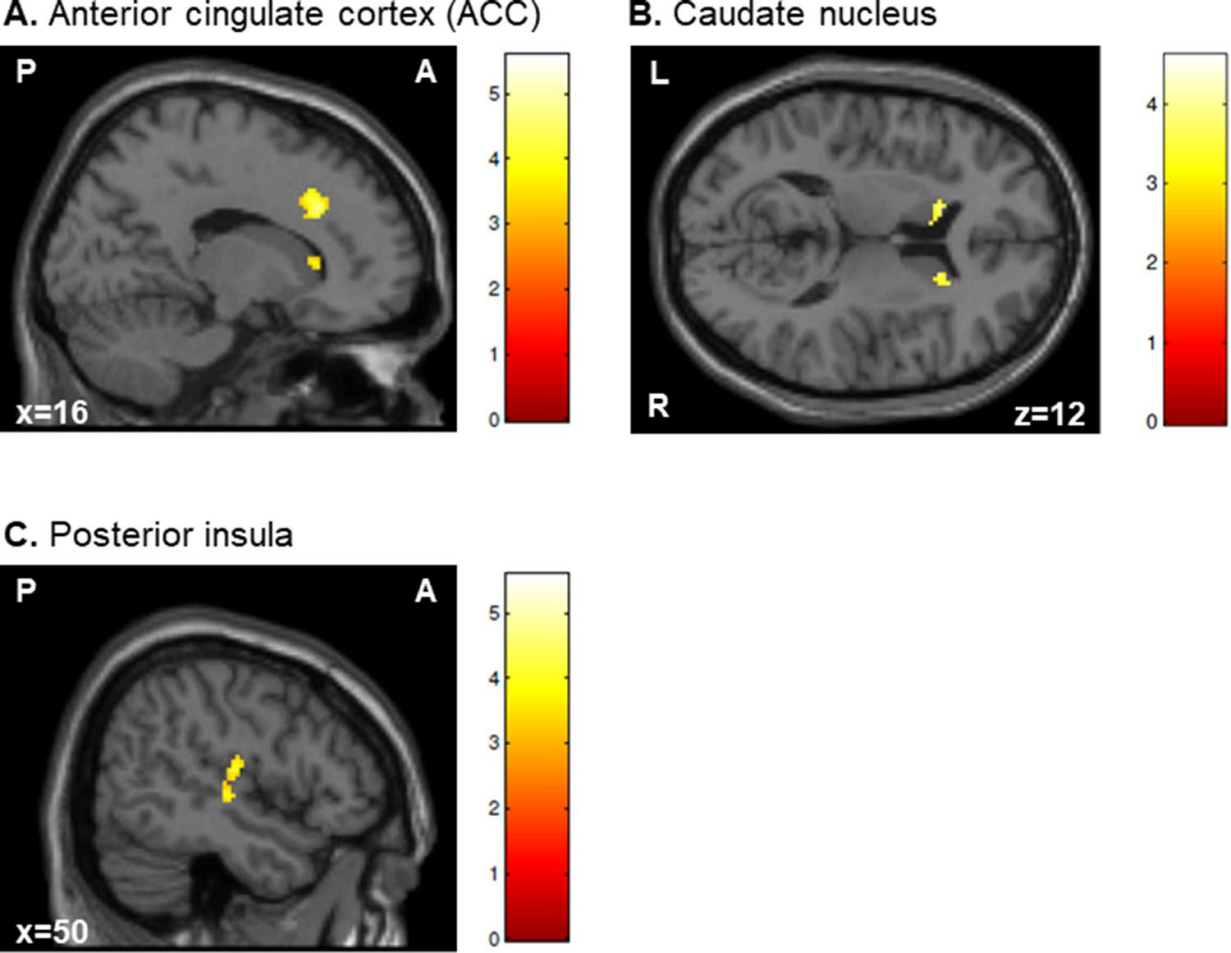
Brain activation maps of the factorial analysis for the contrast *incongruent* > *congruent* (i.e., activations associated with the Simon effect) for the healthy young subjects (n = 13). Incongruent trials compared to congruent trials (i.e., the *overall* Simon effect) induced significant activation clusters in the right anterior cingulate cortex (ACC; panel **A**), the caudate nucleus bilaterally (panel **B**), and the right posterior insula (panel **C**). Note that in panel **A**, the activation cluster in the right caudate nucleus is also visible. All activations are significant at *p* < 0.05, family-wise error (FWE) corrected at the cluster level using an uncorrected voxel-level threshold of *p* < 0.001. Thresholded statistical parametric maps are overlaid onto sections of the Montreal Neurological Institute (MNI) single-subject T1 template image provided by SPM. Coordinates are given in MNI space. Colors reflect the *T*-values of the corresponding voxels. A = anterior; P = posterior; L = left; R = right.

The main effect of congruency (contrasting incongruent trials against congruent trials, *incongruent* > *congruent*, i.e., the *overall* Simon effect) revealed significant activation clusters in the right anterior cingulate cortex (ACC), the (head of the) caudate nucleus bilaterally, and the right posterior insula (Fig. 3). Beta estimates of this contrast were extracted from the peak voxels in the right ACC and bilateral caudate nucleus. Plots of the mean beta estimates for congruent and incongruent conditions as a function of stimulus location (i.e., ipsilateral and contralateral hemifield) suggest that the main effect of congruency was mainly driven by the differential pattern of neural activity in the contralateral hemifield. However, a similar activity pattern was also present for the ipsilateral hemifield—albeit to a lesser degree (see Supplementary Fig. S1). The reverse contrast, *congruent* > *incongruent*, did not yield any significantly activated clusters. Note that all interaction terms did not reveal any significant activations at the predefined statistical threshold.

**Figure 4:**
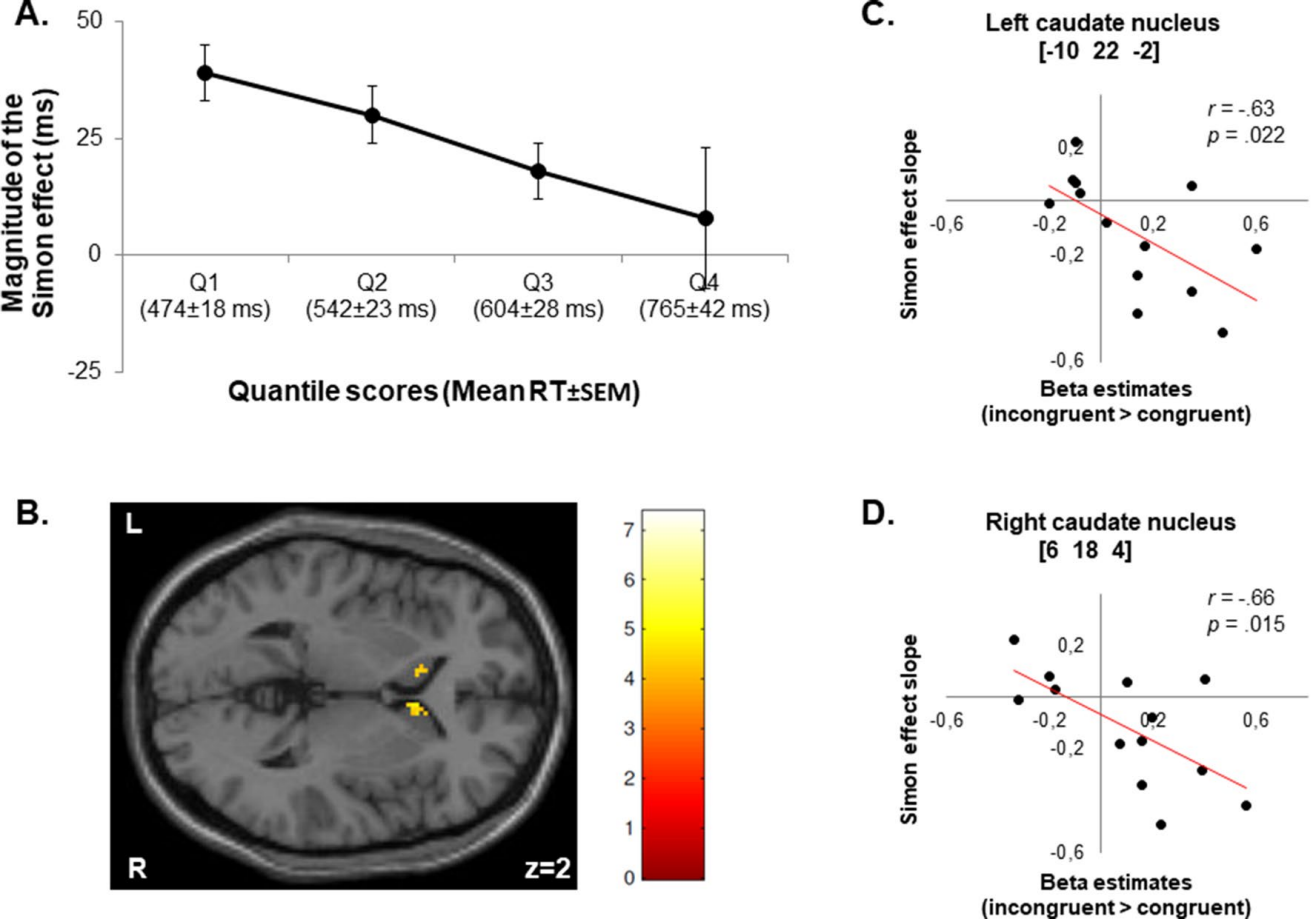
Magnitude of the Simon effect (i.e., the difference in RTs between incongruent and congruent conditions) as a function of response latency (collapsed across responding hands) and brain activation patterns for the corresponding fMRI regression analysis with the RT distribution parameter of selective inhibition (i.e., the course of the Simon effect across the RT distribution as indexed by the slope) as a covariate for the healthy young subjects (n = 13). (**A**) Behavioral results of the response time (RT) distributional analysis: The magnitude of the Simon effect linearly decreased across the RT distribution. For each of the four quantiles (Q1–Q4) the respective mean RT and standard error of the mean (SEM) are given (in parentheses). Error bars indicate standard errors of the mean Simon effects (SEM_SE_). (**B**) Mean activation map of the fMRI regression analysis: Across subjects, neural activity in the bilateral caudate nucleus significantly co-varied with the Simon effect slope values (i.e., the course of the Simon effect across the entire RT distribution used as a behavioral index of selective inhibition). Activations are significant, based on small volume correction at *p* < 0.05, family-wise error (FWE) corrected for the search volume using an anatomically defined mask of the dorsal striatum (i.e., caudate nucleus and putamen). The thresholded statistical parametric map is overlaid onto a section of the Montreal Neurological Institute (MNI) single-subject T1 template image provided by SPM. The coordinate is given in MNI space. Colors reflect the *T*-values of the corresponding voxels. L = left; R = right. (**C**) & (**D**) Scatterplots depicting the correlation between the individual Simon effect slopes across the entire RT distribution (*y*-axis) and the beta estimates for the contrast incongruent > congruent extracted from the peak voxels of the clusters in the left caudate nucleus (C; *x*-axis) and right caudate nucleus (D; *x*-axis): Higher activations in the left and right caudate nucleus were associated with more negative Simon effect slopes. The numbers in square brackets indicate the respective *x*-, *y*-, and *z*-coordinates in MNI space.

At the whole-brain level, the fMRI regression analysis for the contrast of incongruent versus congruent trials with the Simon effect slopes (i.e., the course of the Simon effect across the entire RT distribution) as a covariate did not reveal significant activations at the predefined threshold of *p* < 0.05 FWE-corrected for multiple comparisons at the cluster level (cluster-forming threshold *p* < 0.001 uncorrected). When the cluster-defining threshold was lowered to *p* < 0.005, the regression analysis yielded significant activations (only) within the bilateral striatum (particularly the caudate nucleus) at *p* < 0.05, FWE-corrected at the cluster level. The fMRI regression analysis showed a significant association between the Simon effect slope values and activity in the caudate nucleus bilaterally across subjects (Table 2; Fig. 4B), after applying small volume correction for the dorsal striatum (i.e., caudate nucleus and putamen). This result was corroborated by negative correlations between the beta estimates for the contrast incongruent > congruent extracted from the peak voxels of the activated clusters in the left and right caudate nucleus and the individual Simon effect slopes for the entire RT distribution. That is, higher activations in the left and right caudate nucleus were associated with more negative Simon effect slopes across the entire RT distribution (Fig. 4C,D). For further fMRI regression analyses on the separate Simon effect slopes between each of the four RT quantiles (i.e., for the fast, middle, and slow RT segments), please refer to the Supplement (Supplementary Analysis and Supplementary Fig. S2).

**Table 2:** Brain activation results of the fMRI regression analysis with the RT distribution parameter of selective inhibition (i.e., the decrease of the Simon effect across the RT distribution as indexed by the slope) as covariate (for the contrast *incongruent* > *congruent*).

	Hemisphere	Voxels	Max *T*-value	MNI coordinates		
				x	y	z
Caudate nucleus	R	47	5.32	6	18	4
	L	42	7.35	− 10	22	− 2

A (sub)maximum with the corresponding coordinates in Montreal Neurological Institute (MNI) space, *T*-value, and the number of voxels are given.

Activations are significant, based on small volume correction at *p* < 0.05, family-wise error (FWE) corrected for the search volume using an anatomically defined mask of the dorsal striatum (i.e., caudate nucleus and putamen).

L = left; R = right.

## Discussion

The present study aimed to delineate further the neural mechanisms underlying the control of response interference. In particular, we probed the hypothesis that striatal activity is associated with individual differences in the efficiency of selective inhibition engaged in controlling response interference. For that purpose, healthy (young) subjects underwent fMRI scanning during the performance of a unimanual Simon task. To focus on the neural mechanisms of the selective inhibition process, a specific theory-derived behavioral index of the (individual) efficiency of selective inhibition (derived from RT distribution analysis; outlined above) was regressed with functional imaging data across subjects^71^.

In line with previous studies that used a similar version of the unimanual Simon task^45,72^, behavioral results indicated a significant asymmetrical Simon effect. Thus, the current unimanual Simon task successfully elicited a stimulus–response conflict, which was more pronounced in the contralateral hemifield (i.e., in the visual field contralateral to the responding hand). Furthermore, an RT distributional analysis showed a significant decrease in the Simon effect as individual RTs increased, reflecting the process of selective inhibition^51^.

Notably, the decrease in the (mean) Simon effects across the RT distribution seemed mostly be driven by ipsilateral stimuli (i.e., stimuli in the visual field on the side of the responding hand). According to the activation-suppression hypothesis^49^, the decreasing Simon effect with increasing (intra-individual) RTs for ipsilateral stimuli might indicate that selective inhibition was more efficient in resolving interference in the ipsilateral hemifield, resulting in a markedly reduced overall Simon effect (in contrast to the contralateral hemifield).

It has been proposed that factors such as the spatial position of the effectors (responding hand or finger), the (relative) stimulus location within a visual field, the individual’s handedness, and the hemispheric lateralization of processes involved in the Simon task (e.g., motor attention, response selection, interference processing) impact the resolution/magnitude of the Simon effect^73^. Indeed, left–right asymmetries in the mean Simon effect (i.e., more substantial Simon effects on one side than on the other) were shown to occur robustly in *bimanual* tasks^74,75^. In contrast, in *unimanual* experimental set-ups, the effects of response-related factors on asymmetries in the mean Simon effect have been less conclusive^76^. As noted above, asymmetrical Simon effects between hemifields were found in two previous behavioral studies that used a unimanual Simon task, with smaller mean Simon effects on the side of the responding hand^45,72^. Still, other fMRI studies using unimanual variants of the Simon task did not report asymmetries in task performance^39,77–79^. To our knowledge, there are currently no studies available that analyzed the time course of the Simon effect across the RT distribution as a function of lateralized visual stimuli (neither for bimanual nor unimanual response set-ups). The current asymmetric pattern of behavioral results might be accounted for by a processing advantage towards the side of the responding hand (i.e., here in the ipsilateral hemifield; for similar accounts refer to ^72,76^). In line with this assumption, previous research suggested that cognitive control is enhanced near the hands, as indicated by reduced interference effects for stimuli (re)presented close to the responding hand^80^.

Whole-brain functional imaging analyses revealed that the *overall* Simon effect (i.e., the contrast between incongruent and congruent conditions independent of responding hand and hemifield) induced increased neural activation in the (right) ACC, (right) posterior insula, and the caudate nucleus bilaterally. Crucially, an ROI-based fMRI regression analysis confirmed the notion that more efficient selective inhibition (reflected in a more pronounced decrease of the Simon effect across the entire RT distribution) was associated with increased activation in the caudate nucleus bilaterally.

A specific contribution of the dorsal striatum to the control of response interference has been emphasized by studies that reported impairments in resolving response interference in patients with neurodegenerative diseases affecting the striatum, such as Parkinson’s disease (PD)^37^. Furthermore, the current results add to previous findings in both brain lesion^38,45^ and neuro-computational modeling studies^33^ that applied (variants of) the Simon task by providing support for a (functional) role of the dorsal striatum (particularly the caudate nucleus) in selective inhibition. Several previous functional imaging studies have also implicated that the striatum is engaged during conditions that entail the anticipation^81–83^ and (subsequent) preparation of selective inhibitory control^84,85^. Specifically, these studies applied a selective stop-signal task in which subjects were instructed to initiate two responses (which should be executed in go-trials) and to suppress one particular response while continuing the other in case of a stop-signal. Additionally, information on which particular response (out of the two) might need to be selectively inhibited was given at the start of each trial.

Beyond these findings, global (non-selective) response inhibition functions have frequently been related to the right IFG and its interconnections to subcortical structures, such as the STN, during withholding or canceling an inappropriate response in standard go/no-go and stop-signal tasks, respectively^19,20^. Notably, it has been suggested that the control process concerning (conflict-driven) inhibitory demands subtly differ between *selective* inhibition in response interference control (as assessed by the Simon task) and *global* inhibition in response inhibition (as assessed by go/no-go or stop-signal tasks)^86^. Consequently, both control processes (i.e., selective and global inhibition) are thought to be implemented by distinct, albeit partly overlapping, cortical and subcortical neural networks^87,88^. It has been proposed that the Stroop, Eriksen flanker, and Simon tasks share the need to control a (prepotent) response tendency activated by a task-irrelevant stimulus dimension to execute another response based on a task-relevant stimulus feature^2^. Still, there is an ongoing debate concerning the (shared) cognitive control processes that may underlie these interference tasks—and whether they share a common mechanism of selective inhibition (for detailed studies on this issue see for example^89–92^). Latent-variable factor analyses across a variety of response inhibition/interference tasks implied closely related^93^ but dissociable forms of inhibition in the Stroop, Eriksen flanker, and Simon tasks (among other paradigms)^94,95^.

Taken together, by utilizing a theory-derived behavioral index of selective inhibition in the Simon task^49,51^, the present results suggest a functional contribution of the dorsal striatum (particularly the caudate nucleus) to the efficiency of the selective inhibition process engaged in the control of response interference. It should be noted that the current findings explicitly rely on the assumption that controlling stimulus–response interference in the Simon task entails the selective inhibition of interfering response tendencies^96,97^ and that this selective inhibition process is revealed by a decrease of the Simon effect magnitude across the individual RT distribution^51^. Indeed, decreasing Simon effects with longer individual RTs have been reported in several previous studies on response interference control in healthy subjects^98,99^ and empirically tied to (efficient) selective inhibition^48^. Furthermore, even though the interference in the Simon task might also result from a failure in (selective) attentional control^100^, several findings suggest that its resolution mainly occurs during the response selection/inhibition process (i.e., at the level of the response execution)^101^, rather than at the perceptual level during stimulus encoding^86,102^.

However, an alternative account for the decrease in the magnitude of the Simon effect with longer (intra-individual) RTs proposed that the automatic response activation by the (task-irrelevant) stimulus location spontaneously decays over time, leading to reduced interference (and hence a reduced Simon effect) with slower responses^103,104^. More recently, an elaborated diffusion process model has been introduced to predict the commonly observed decreasing Simon effects across the RT distribution^105^. More specifically, this *diffusion model for conflict tasks* (DMC) formally specifies the mechanisms underlying the Simon effect by modeling the time course of the automatic (task-irrelevant) response activation as a brief pulse-like function (while assuming the controlled processing channel driven by the task-relevant stimulus feature to constantly input into a diffusion process as long as the stimulus is present). Consequently, the output of such a brief pulse-like activation should affect short RTs more strongly than longer ones, resulting in the typically more substantial Simon effect with relatively faster responses, which then becomes reduced across the RT distribution^105,106^. It should be noted here that the mathematical model does not specify whether a decrease in automatic response activation might be due to an active inhibition process^49^ or spontaneous decay^103^.

On the other hand, decreasing Simon effect functions might reflect cognitive processes beyond what is expected based on a diminished impact of (task-irrelevant) response activation with slower responses—either due to an active process of selective inhibition or passive decay. To reveal (putative) alternative and/or additional explanations of decreasing Simon effects, in a recent study manipulations of different stages of cognitive processing (i.e., perceptual, decision, and motor execution) that might modulate the Simon effect were combined with distributional analyses^107^. For example, prolonging the duration of the decision process (by increasing the number of stimulus–response (S–R) pairs) resulted in smaller (overall) Simon effects with four S–R pairs (for which mean RTs were also longer) than with two S–R pairs. Importantly, within each of the two conditions, the Simon effect also decreased with slower (individual) responses. In other words, at any given time point of the RT distribution, the Simon effect was smaller with four S–R pairs than with two S–R pairs—even when controlling for the different overall RTs between conditions. Since the time was controlled for in which the impact of the (task-irrelevant) response activation diminished (either due to active inhibition or passive decay), the authors proposed that the strength of the task-irrelevant activation (i.e., the response tendency evoked by the stimulus location) may have been weakened a priori in the four-stimulus condition compared to the two-stimulus condition, which in turn reduced the (overall) magnitude of the Simon effect^107^. While processing the (task-irrelevant) stimulus location should not systematically vary in the current version of the Simon task, it might still be subject to individual variability. Consequently, the observed decrease in the magnitude of the Simon effect across the RT distribution might be attributed to person-related changes in the time course (i.e., onset, build-up rate) and/or strength of the (automatic) response activation, the controlled selective inhibition of that response activation, or both.

Moreover, the current ACC finding is consistent with other neuroimaging studies that commonly reported ACC activations related to (response) conflict detection during a wide range of (conflict-driven) inhibitory control tasks^16^. Since a pronounced stimulus–response conflict characterized the incongruent conditions (compared to the congruent conditions) in the current unimanual Simon task, our results provide further support for the involvement of the ACC in the detection and/or monitoring of response-related conflict.

Besides, the current functional imaging results revealed that response interference in incongruent conditions (compared to congruent conditions) activated a cluster in the (right) posterior insula. Although less often mentioned than prefrontal regions, the insular cortex has also been suggested to play a role in cognitive control processes^12,108^. In this regard, the insula is proposed to be (more generally) involved in the maintenance of task sets across trials^109^, the estimation of forthcoming control demands^27^, and the initiation and adjustment of appropriate attentional control mechanisms to external stimuli^110^. Moreover, there is converging evidence that the insula and the ACC are functionally related, forming an “attentional” network that initiates control processes in response to conflict^111^. In the same vein, the present results support a role of the insula (together with the ACC) in response conflict, albeit its (specific) function in the control of response interference warrants further investigation.

The relatively small sample size can be considered a limitation of the current study. However, our study does not seem to be underpowered since we observed significant activations at standard cluster-corrected thresholds for the contrast between incongruent and congruent conditions^67^. Also, the argument that our current findings might be difficult to be reproduced falls short, since the current activations nicely confirm (and also extend) previous studies that found similar activations in cortical (and subcortical) regions when investigating response interference control with the Simon task with comparable sample sizes^39,40,78,112,113^ as well as with larger ones^79,114–116^. In contrast, some functional imaging studies examining similar-sized groups of healthy participants did not find significant results for the comparison of incongruent and congruent conditions in the Simon task^53,54,58^. These discrepant findings might be partly due to procedural differences between the studies (e.g., length of inter-trial intervals, presentation of a congruency cue) that potentially affected the perceived response interference and, in turn, the associated neural processes^117^.

## Conclusions

The present functional imaging results revealed a fronto-striatal network supported by the insula during response interference control. Most importantly, the dorsal striatum (particularly the caudate nucleus) substantially contributed to the selective inhibition process engaged in resolving response interference.

## Supplementary information

Supplementary Information.
