# Supplementary material for: Control of response interference: caudate nucleus contributes to selective inhibition
Source: Sci Rep. 2020 Dec 1;10:20977. doi: 10.1038/s41598-020-77744-1 (PMC7708449; doi:10.1038/s41598-020-77744-1)
Supplement: Supplementary file 1 — Supplementary Information. [file 41598_2020_77744_MOESM1_ESM.docx]

**Supplementary Material**

**Control of response interference:**

**caudate nucleus contributes to selective inhibition**

Claudia C. Schmidt^*^, David C. Timpert, Isabel Arend, Simone Vossel, Gereon R. Fink, Avishai Henik, & Peter H. Weiss

*** Correspondence:** Claudia Schmidt: [c.schmidt@fz-juelich.de](mailto:c.schmidt@fz-juelich.de)


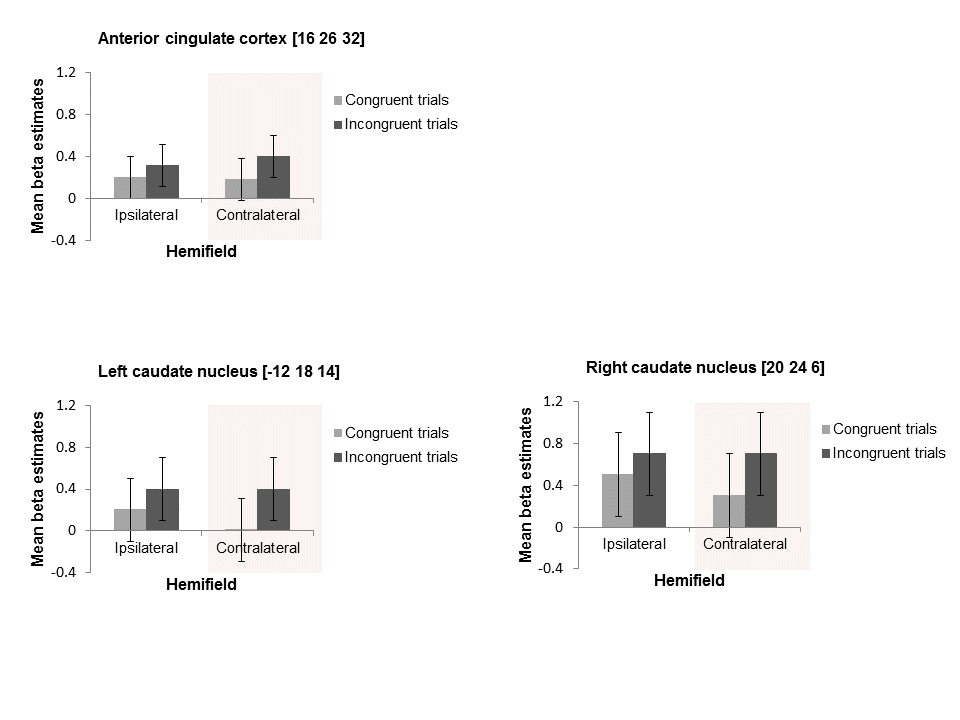


**Supplementary Figure S1. Mean beta estimates as a function of stimulus location and stimulus-response congruency for the peak voxels in the right anterior cingulate cortex (ACC) and the bilateral caudate nucleus.**

The observed pattern of neural activity in the ACC and the caudate nucleus suggests that the main effect of congruency was mainly driven by the larger activity differences between incongruent and congruent trials in the contralateral hemifield (compared to those differences in the ipsilateral hemifield). However, formal testing of these mean beta estimates using a 2 x 2 repeated measures ANOVA revealed that the interaction effects of stimulus location (ipsilateral hemifield, contralateral hemifield) and congruency (congruent, incongruent) were not significant (all *p* > .181).

Error bars indicate standard errors of the mean (SEM). The numbers in square brackets indicate the respective *x*-, *y*-, and *z*-coordinates in MNI space.

**Supplementary Analysis.** Functional magnetic resonance imaging (fMRI) regression analyses with the separate Simon effect slopes between each of the four response time (RT) quantiles (i.e., for the fast, middle, and slow RT segments) as covariates (for the contrast *incongruent* > *congruent*).

We also extracted separate Simon effect slopes between each of the four RT quantiles and included each of the three slopes as covariates in further fMRI regression analyses. Based on the *activation-suppression hypothesis* (van den Wildenberg et al., 2010), a decrease of the Simon effect with slower responses (i.e., a negative-going slope across the RT distribution) reflects the process (and efficiency) of selective inhibition. Accordingly, significant activations in the fMRI regression analysis should indicate brain regions that co-varied with the individual efficiency of selective inhibition.

Similar to the analysis described in the main manuscript, for each participant, the separate slopes accounting for the change of the Simon effect between the first and second quantile (slope 1; fast RT segment), between the second and third quantile (slope 2; middle RT segment), and between the third and fourth quantile (slope 3; slow RT segment) were computed based on ordinary least squares (OLS) regression. The three slopes were then included as covariates in separate fMRI regression analyses with the differential contrast images for the main effect of congruency (incongruent > congruent). Significance testing was performed on each covariate separately using a directed *t*-contrast, and activations were considered significant for a threshold of *p* < .05, family-wise error (FWE) corrected at the cluster level using an uncorrected voxel-level threshold of *p* < .001.

Results of these fMRI regression analyses did not yield any significant activations for the Simon effect slopes between the first and second quantiles (slope 1) or between the second and third quantiles (slope 2). In contrast, the slope for the slowest RT segment (i.e., between the third and fourth quantile) significantly co-varied (only) with activation in the bilateral caudate nucleus (*right*: peak voxel MNI coordinates: *x* = 4, *y* = 16, *z* = 4; maximal *t*-value = 7.59; *left*: peak voxel MNI coordinates: *x* = -10, *y* = 22, *z* = -2; maximal t-value = 5.45).

To further illustrate the mean activation pattern, we calculated separate Pearson correlations between the beta estimates for the contrast incongruent > congruent extracted from the activated peak voxels in the left and right caudate nucleus and the three Simon effect slopes. There was a significant correlation between the beta estimates in the left and right caudate nucleus and the third Simon effect slope (Supplementary Fig. 2; last column), but not between the mean beta estimates in the left and right caudate nucleus and the first Simon effect slope (Supplementary Fig. 2; first column) or the second Simon effect slope (Supplementary Fig. 2; middle column).

Taken together, (increased) activation in the bilateral caudate nucleus specifically co-varied with the Simon effect slope in the slowest RT segment (i.e., slope 3), indicating that the process (and efficiency) of selective inhibition may be particularly revealed in the slowest part of the RT distribution.


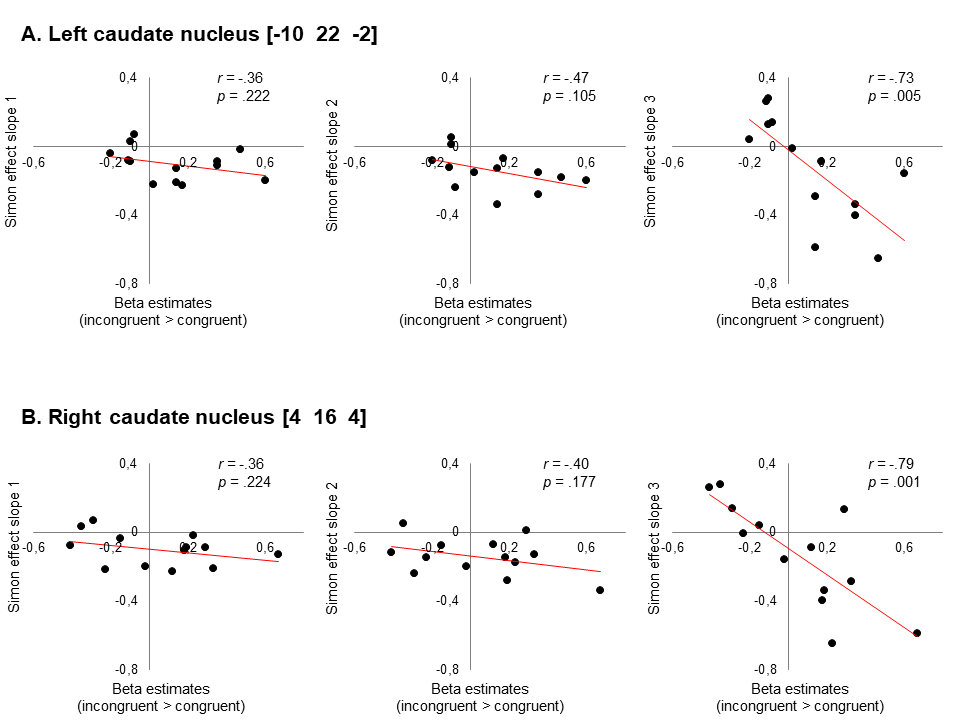


**Supplementary Figure S2. Brain activation patterns for the fMRI regression analyses.**

Scatterplots depicting the correlation between the individual Simon effect slopes between each of the RT quantiles (i.e., slope 1, slope 2, and slope 3; *y*-axis) and the beta estimates for the contrast incongruent > congruent extracted from the peak voxels in the left caudate nucleus (A; *x*-axis) and the right caudate nucleus (B; *x*-axis). The numbers in square brackets indicate the respective *x*-, *y*-, and *z*-coordinates in MNI space.

Reference:

van den Wildenberg, W. P. M., Wylie, S. A., Forstmann, B. U., Burle, B., Hasbroucq, T., & Ridderinkhof, K. R. (2010). To head or to heed? Beyond the surface of selective action inhibition: a review. *Frontiers in Human Neuroscience, 4*, 222.
